# Lambeau de Mac Gregor sur ring finger chez un enfant

**DOI:** 10.11604/pamj.2022.41.151.33501

**Published:** 2022-02-21

**Authors:** Mohamed Amine Oukhouya, Hind Cherrabi

**Affiliations:** 1 Service de Chirurgie Pédiatrique, Faculté de Médecine et de Pharmacie d’Agadir, Université Ibn Zohr, Centre Hospitalier Universitaire Souss Massa, Agadir, Maroc

**Keywords:** Ring finger, enfant, lambeau de Mac Gregor, Ring finger, child, Mac Gregor flap

## Abstract

We report the case of an 11-year-old female child who injured her ring finger (ring on the 4^th^ finger hung on a nail fixed to the wall during a jump). Clinical examination showed 4^th^ finger degloving injury with exposure of bone and tendon system and 3^rd^ phalanx amputation (A). Following consultation with the family, the finger was preserved and McGregor inguinal flap was performed for aesthetic and functional outcome. Emergency surgery was performed under general anesthesia, with the patient in a supine position and preparation of the right upper limb and right flank. McGregor inguinal flap was performed after anatomical identification of the flap to place on the pathway of the superficial iliac artery (B). After dissecting the flap, it was tubularized on the finger to cover it (C). The postoperative course was uneventful and after three weeks the flap was weaned under general anesthesia (D); the result after one month was very satisfactory (E).

## Image en médecine

Nous rapportons le cas d´un enfant de sexe féminin agé de 11 ans victime d´un traumatisme type ring finger (bague du 4^e^ doigt accrochée à un clou fixé au mur lors d´un saut), l´examen clinique montre un dégantage du 4^e^ doigt avec mise à nue de l´os et du système tendineux et amputation de la 3^e^ phalange (A), après concertation avec la famille on a opté pour la préservation du doigt et la réalisation d´un lambeau inguinal de Mac Gregor, cette intervention avait pour but de préserver le doigt tant sur le plan esthétique et fonctionnel. L'intervention a été réalisée en urgence sous anesthésié générale, avec installation du patient en décubitus dorsal et préparation du membre supérieur droit et du flanc droit. On a prélevé un lambeau inguinal de Mc Gregor après avoir fait un repérage anatomique du lambeau qui doit siéger sur le trajet de l'artère iliaque superficielle (B), après avoir disséqué le lambeau on l'a tubulisé sur le doigt pour le couvrir (C). Les suites opératoires étaient simples, et après trois semaines on a procédé au sevrage du lambeau sous anesthésie générale (D), le résultat après un mois est très satisfaisant (E).

**Figure 1:**
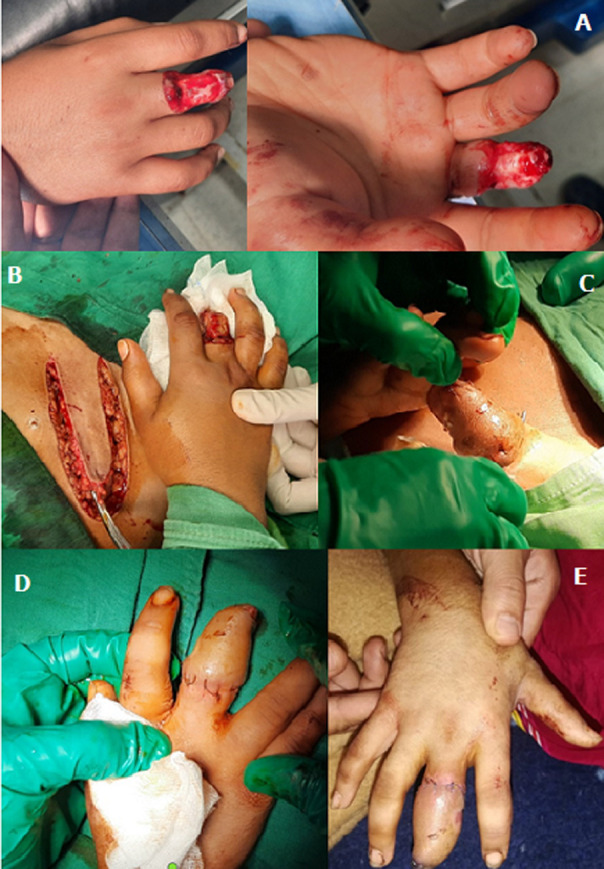
aspect clinique du ring finger (A); image opératoire du lambeau inguinal (B); tubulisation du lambeau sur le doigt (C); résultat après sevrage du lambeau (D); résultat après un mois (E)

